# Evaluating cognitive performance using the National Institutes of Health Toolbox Cognitive Battery in children with traumatic brain injury

**DOI:** 10.1017/S135561772510146X

**Published:** 2025-06

**Authors:** Bailey Petersen, Ngoc-Thanh N. Vo, Nivinthiga Anton, Keith Owen Yeates, Amery Treble-Barna

**Affiliations:** 1 Department of Physical Medicine and Rehabilitation, University of Pittsburgh School of Medicine, Pittsburgh, PA, U.S; 2 Department of Psychology, Alberta Children’s Hospital Research Institute, and Hotchkiss Brain Institute, University of Calgary, Calgary, AB, Canada

**Keywords:** Traumatic, brain injury, child, neuropsychology, outcomes, recovery

## Abstract

**Objective:**

We examined cognitive performance in children with complicated mild-severe traumatic brain injury (TBI) versus orthopedic injury (OI) using the National Institutes of Health Toolbox Cognitive Battery (NIH TB-CB).

**Method:**

We recruited children ages 3–18, hospitalized with complicated mild-severe TBI (*n* = 231) or orthopedic injury (OI, *n* = 146). Cognition was assessed using the NIH TB-CB at six and twelve months post-injury. We used linear mixed models to assess associations of injury group (TBI versus OI), timepoint (six versus twelve months), and the interaction of injury group and timepoint with NIH TB-CB Total Cognition, Fluid Cognition, and Crystallized Cognition composites, adjusted for sex and socioeconomic status (SES), with Bonferroni correction. We evaluated differences in cognition stratified by injury severity (complicated mild–moderate TBI vs severe TBI) using ANCOVA, adjusting for sex and SES.

**Results:**

Neither injury group nor the interaction of group and timepoint were associated with Total (group: *p* = 0.50; timepoint*group: *p* = 0.185), Fluid (group: *p* = 0.297; timepoint*group: *p* = 0.842), or Crystallized Cognition (group: *p* = 0.039; timepoint*group: *p* = 0.017). However, children with severe TBI performed significantly worse on Fluid and Total Cognition than children with complicated mild–moderate TBI at six months (Fluid: *p* = 0.004, partial *η*^2^ = 0.06, moderate effect, Total: *p* = 0.012 partial *η*^2^ = 0.03, small–moderate effect) and twelve months post-injury (Fluid: *p* < 0.001, partial *η*^2^ = 0.11, moderate–large effect, Total: *p* = 0.002, partial *η*^2^ = 0.06, moderate effect).

**Conclusions:**

The NIH TB-CB detects worse cognitive functioning in children with severe TBI six-twelve months post-injury, largely driven by differences in Fluid Cognition. Our findings suggest the NIH TB-CB may be suitable for monitoring cognition in children with TBI.

## Statement of Research Significance

**Research Question(s) or Topic(s):** Can the National Institutes of Health Toolbox Cognitive Battery be used to assess cognitive recovery in children with complicated mild-severe traumatic brain injury compared to orthopedic injury? Can this assessment detect differences between children with complicated mild-moderate and children with severe traumatic brain injury? **Main Findings:** The Toolbox Cognitive Battery detects lower cognitive performance in children with severe traumatic brain injury at six and twelve months post-injury, but not children with complicated mild–moderate traumatic brain injury. These differences occur largely in Fluid Cognition and are most pronounced in children with severe traumatic brain injury. **Study Contributions:** This assessment has not been used to compare cognitive recovery for children with complicated mild-severe traumatic brain injury to children with orthopedic injury (OI). In settings without the resources for extensive neuropsychological testing, the Toolbox Cognitive Battery may be a suitable option for monitoring cognition in children with traumatic brain injury.

## Introduction

Pediatric traumatic brain injury (TBI) is a leading cause of childhood morbidity and mortality in the United States (Li & Liu, 2013). Up to 50% of children with TBI experience lasting cognitive impairments that can affect their ability to function at home, at school, and in the community (Keenan et al., 2021; Treble-Barna et al., 2017; Treble-Barna et al., 2017a). Cognitive recovery after TBI in children has been well characterized using a plethora of neuropsychological tests (Goh et al., 2021). However, many of these tests take a long time to administer in both research and clinical settings and require administration by a trained neuropsychologist or neuropsychological technician. Many settings do not have the resources, personnel, or infrastructure to support comprehensive neuropsychological evaluation for children throughout all stages of TBI recovery (Bailey et al., 2022). In these settings, assessing cognitive recovery longitudinally currently poses a substantial challenge for clinical researchers and clinicians alike (Bailey et al., 2022). In addition, selection of tests can vary widely across domains of cognition and across age ranges, and heterogeneity in test selection makes comparison across studies difficult. The field of pediatric TBI would benefit from a brief but comprehensive test battery that can be performed by individuals without rigorous training in the selection and administration of neuropsychological tests and used to evaluate cognition in children across the spectrum of recovery after TBI.

The National Institutes of Health Toolbox for Assessment of Neurological and Behavioral Function (NIH TB) was developed to assess behavior across a series of functional domains in a large-scale census-based study (Gershon et al., 2010, 2013). The NIH TB Cognitive Battery (NIH TB-CB) is a performance-based assessment of cognition spanning ages 3 – 85 years (Taylor et al., 2022; Weintraub et al., 2013). This battery provides age-corrected standardized scores normalized to typically developing children and adults. The NIH TB-CB combines a series of cognitive tests that span seven domains of cognition (executive function, attention, episodic memory, language, reading, processing speed, and working memory). The full battery can be used for children 7 years and older and provides 3 composite scores: Total Cognition, Crystallized Cognition, and Fluid Cognition. An early childhood version of the NIH TB-CB can be used for children ages 3 – 6 to provide a measure of Early Childhood Cognition. In this study, we used the second version of the NIH TB-CB, available since 2015, which is administered portably on a tablet.

Since its debut, the NIH TB-CB has been used in clinical research across a wide variety of neuropsychological conditions, including adults with brain injury (Carlozzi et al., 2017; Tulsky et al., 2017; Tulsky et al., 2017) and children with uncomplicated mild TBI/concussion (Chadwick et al., 2021). The NIH Common Data Elements developed in 2012 for pediatric TBI listed the NIH TB-CB as an emerging measure of neurobehavioral impairment (McCauley et al., 2012). In a small heterogeneous sample of children with acquired brain injury, the NIH TB-CB was shown to be feasible to administer in inpatient rehabilitation settings and day treatment rehabilitation facilities (95% of children tested were able to complete the test); while the study was underpowered to determine statistical differences in cognition, children with ABI had poorer performance on fluid cognition than crystallized cognition (Watson et al., 2020). Another study of children with mild TBI reported poorer Fluid Cognition on the NIH TB-CB relative to children with OI in the ten days following injury, but that the groups were comparable at three and six months post-injury (Chadwick et al., 2021). These findings are consistent with the vast literature using traditional neuropsychological tests demonstrating that Fluid Cognition is worse after TBI, while Crystallized Cognition is often spared (Tulsky, Carlozzi et al., 2017). Other studies have used the NIH TB-CB in children with TBI to assess cognition in a variety of contexts. It has been used as a measure of cognition in a study of driving skills in adolescents following mild TBI (Rivara et al., 2023) and in a study of different learning styles in children after mild TBI (Lundine et al., 2018), and to determine cognitive response to treatment with virtual reality cognitive behavioral therapy in children with complicated mild-severe TBI (Shen et al., 2024). Despite its widespread use, NIH TB-CB performance for children with complicated mild-severe TBI has not been compared to OI. Additionally, NIH TB-CB performance has not been compared between children with milder TBI (complicated mild–moderate TBI) and those with severe TBI.

In this study, we compared cognitive performance in children with complicated mild through severe TBI to children with OI using the NIH TB-CB at six and twelve months post-injury. We hypothesized that children with TBI would have poorer cognitive performance on the NIH TB-CB than children with OI, and that children with severe TBI would have poorer cognitive performance than children with complicated mild–moderate TBI. Furthermore, we hypothesized that differences would be driven by poorer performance on Fluid Cognition.

## Methods

### Participants

Participants were drawn from the ongoing Epigenetic Effects on Pediatric TBI Recovery (EETR) study (Treble-Barna et al., 2020) at the UPMC Children’s Hospital of Pittsburgh, completed in accordance with the Helsinki Declaration with approval from with the University of Pittsburgh’s Institutional Review Board. EETR is an observational, prospective, longitudinal concurrent cohort study of children aged 3 – 18 years hospitalized overnight for complicated mild to severe non-penetrating TBI or OI. We selected children with OI as a comparison group to control for the experience of being hospitalized with a traumatic injury and to adjust for pre-morbid risk factors such as lower socioeconomic status (SES) that are associated with an increased risk for sustaining a traumatic injury (Asarnow et al., 1995; Yeates, 2010).

Complicated mild TBI is defined as a lowest post-resuscitation Glasgow Coma Scale (GCS) score (Teasdale & Jennett, 1974) of 13 – 15 with clinical neuroimaging indicating intracranial injury; moderate TBI is defined as GCS score of 9 – 12; severe TBI is defined as GCS score of 3 – 8. The OI group includes children with bone fractures, excluding skull or facial fractures, and no signs of head trauma or brain injury (nausea/vomiting, headache, loss of consciousness, GCS score < 15 at any point) or any suspicion of TBI in the electronic medical record. Exclusion criteria for both groups include: (a) non-English-speaking child or parents/guardians; (b) documented or parent-reported history of previous TBI or concussion requiring overnight hospitalization; (c) pre-injury neurological disorder or intellectual disability; (d) pre-injury psychiatric disorder requiring hospitalization; (e) spinal cord injury; (f) sensory or motor impairment precluding study measure completion; or (g) pregnancy at the time of enrollment.

Study staff screen the electronic medical record for potentially eligible children and approach families during their stay in the acute trauma unit. We obtain informed consent from parents/guardians and any participants aged 18 years who are capable of consenting. We also obtain study assent from children aged 8 years or older prior to enrollment. Parents complete questionnaires detailing comprehensive injury, psychosocial, and neurobehavioral function, and children complete the NIH TB-CB and provide blood and saliva biospecimens, with all data collected acutely and at six and twelve months post-injury. We attempted to complete the NIH TB-CB while children were hospitalized acutely; however, very few acute assessments were completed because of time constraints and lack of a setting suitable for testing.

### NIH Toolbox Cognitive Battery

Trained research staff administered the NIH TB-CB. The NIH TB-CB contains seven tests (Table 1). Primary outcomes (dependent variables) were composite scores of the NIH TB-CB: Fluid Cognition (ages 7+ only), Crystallized Cognition (ages 7+ only), and Total Cognition (composite of Fluid Cognition and Crystallized Cognition). Fluid cognition reflects the capacity for learning, problem solving, and processing in novel environments (Akshoomoff et al., 2013). Crystallized cognition reflects the cumulative gathering and storing of knowledge from past learning experiences (Akshoomoff et al., 2013). For children ages 3 – 6, the only composite score provided is the Early Cognition Composite Score, an overall measure of Total Cognition for this age group. To evaluate cognition across the age range, we combined the Early Cognition Composite and Total Cognition Composite scores. As a secondary analysis, we examined scores from the individual seven tests (Supplemental Data).

**Table 1.** Tests and composite scores of the NIH Toolbox Cognitive Battery

Test	Construct	Ages 7+ composite score	Ages 3 – 6 composite score
Dimensional change card sort	Cognitive flexibility	Fluid Cognition	Early Cognition Composite
List sorting working memory	Working memory	Fluid Cognition	
Flanker inhibitory control and attention	Inhibitory control and attention	Fluid Cognition	Early Cognition Composite
Picture sequence memory	Episodic memory	Fluid Cognition	Early Cognition Composite
Pattern comparison	Processing speed	Fluid Cognition	
Picture vocabulary	Word comprehension	Crystallized Cognition	Early Cognition Composite
Oral reading recognition	Single word reading	Crystallized Cognition	

For each individual test and composite, the NIH TB-CB provides both age-adjusted scores standardized to normative values for typically developing children or “fully corrected T scores,” with adjustments based on the child’s age, sex, and family socioeconomic status (SES, which includes race/ethnicity). For this study, we used age-corrected standardized scores in accordance with guidance from the American Board of Clinical Neuropsychology and American Academy of Clinical Neuropsychology, which supports eliminating race as a demographic adjustment in neuropsychological assessments (Bodin et al., 2023). Instead, corrections for demographic variables (sex and SES) were made within the statistical models. Our proxy for SES is a composite z-score of each participant’s maternal years of education and the median income for the participant’s census tract based on their residential address (Kurowski et al., 2016; Narad et al., 2019; Wade et al., 2011; Yeates et al., 2010). Sample *z*-scores were obtained for maternal education and median income, and the two scores were averaged to obtain the composite *z*-score.

### Statistical methods

We used linear mixed models to assess associations of injury group (TBI versus OI), timepoint (six months versus twelve months post-injury), and the interaction of injury group and timepoint with NIH TB-CB cognitive performance (Total Cognition, Fluid Cognition, and Crystallized Cognition). Models were adjusted for sex and SES *z*-score. We included random effects for participant to allow for inclusion of participants with missing data at one timepoint. We calculated Cohen’s d effect sizes for differences between groups using sample means and pooled standard deviation (small *d* = 0.2, medium *d* = 0.5, large *d* > 0.8) (Cohen, 2013). We used a significance level of *α* = 0.05/3 = 0.017 after Bonferroni correction for multiple comparisons for primary outcomes.

We also used linear mixed models to compare injury groups on each of the seven individual test scores of the NIH TB-CB (Supplemental Data). Models included associations with injury group, timepoint, and the interaction of group and timepoint. We used a significance level of *α* = 0.05/7 = 0.007 after Bonferroni correction for multiple comparisons. All models were adjusted for sex and SES composite *z*-score.

As an exploratory analysis due to smaller sample sizes within our TBI group, we also evaluated differences in cognitive performance stratified by injury severity (cmmTBI vs sTBI) using an ANCOVA, adjusting for sex and SES. We assessed assumptions of homogeneity of regression slopes and normality of residuals using a regression analysis and Shapiro Wilks test, respectively. Effect sizes are reported as partial *η*^2^. We also used Spearman’s correlation to determine associations between lowest post-resuscitation Glasgow Coma Scale (GCS) score and NIH TB-CB composite measures of cognitive performance within the TBI sample. Effect sizes are reported as ρ. Because this was a secondary analysis with smaller sample sizes, we kept alpha thresholds at *α* = 0.05.

We conducted linear mixed models using SPSS Statistics (IBM, SPSS Inc.) and all other statistics calculations (ANCOVA, Spearman correlation, etc.) and figures with Python 3.10 using the SciPy and StatsModels packages.

## Results

### Participants

The analytic sample for the current report included participants who completed the NIH TB-CB version 2 at six and/or twelve months post-injury through June 6, 2024. The TBI group included 70 children with complicated mild TBI, 16 children with moderate TBI, and 34 children with severe TBI. Because of the low number of patients with moderate TBI, we combined children with complicated mild to moderate TBI (cmmTBI) for analysis (*n* = 86), consistent with current approaches to analyzing cognitive recovery after TBI in both children (Keenan et al., 2018; Keenan et al., 2019; Levin et al., 2008) and adults (Tulsky et al., 2017). There were no differences in age or sex either across injury groups (Table 2) or within the TBI group stratified by severity (cmmTBI vs. sTBI). Within the cmmTBI group, lowest post-resuscitation GCS scores range from 9 to 15, with 81% falling within the complicated mild TBI category.

**Table 2.** Participant demographics

	OI (*n* = 60)	TBI (*n* = 120)			
		sTBI (*n* = 34)	cmmTBI (*n* = 86)	Differences between TBI severity, *p*-value	Differences between injury group, *p*-value
Age at Injury in yrs (median [IQR])	10.61 [6.86, 14.24]	10.84 [6.02, 14.57]	10.93 [6.20, 14.32]	0.71	0.99
Sex, *n* (% male)	41 (68.3)	22(64.7)	53 (61.6)	0.92	0.54
Lowest post-resuscitation GCS (Median [IQR])	---	3.5 [3.0, 7.0]	15.0 [14.0, 15.0]	< 0.001*	
SES Composite *Z*-score (Mean (SD))	0.16 (0.83)	−0.14 (0.69)	0.03 (0.83)	0.28	0.16
Child race, n (%)				0.33	0.20
White	48 (80.0)	28 (82.4)	76 (88.4)		
Black or African American	8 (13.3)	5 (14.7)	6 (7.0)		
Asian	2 (3.3)	1 (2.9)	0 (0)		
Other	0 (0)	0 (0)	0 (0)		

We assessed differences between injury groups (TBI vs OI) and TBI severity groups (cmmTBI vs sTBI) using a *t*-test for normally distributed continuous data (SES Composite Z-score), a Mann–Whitney *U* Test for non-normally distributed continuous data and a chi-squared test for categorical data. Race was categorized as white vs. all other race groups (combined due to small cell sizes). *indicates significant differences (*p* < 0.05).

**Figure 1.**
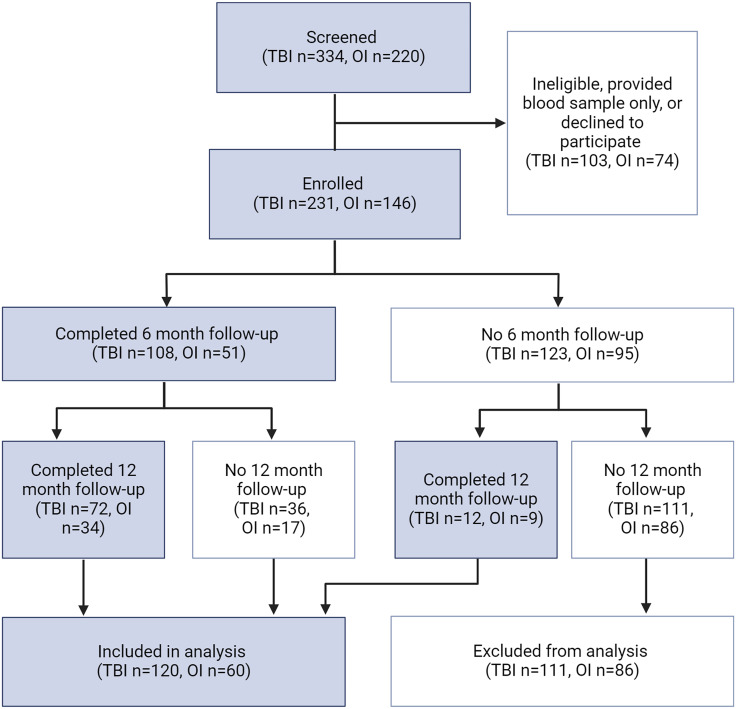
Flow chart of participant enrollment, follow-ups and analysis. Only participants with complete six and/or twelve month outcomes and demographic data. Created with biorender.com.

A subset of these participants had demographic data and NIH TB-CB scores at six months and/or twelve months post-injury (120 children with TBI, 60 children with OI, Figure 1). Despite substantial attrition, children who were included in the analysis did not differ from children who were excluded in demographics or TBI severity (*p* > 0.05, Supplemental Data). Similarly, children who completed only the six month follow-up did not differ from those completing the twelve month follow-up or those completing both follow-ups (*p* > 0.05, Supplemental Data).

### Cognition by injury group

**Figure 2.**
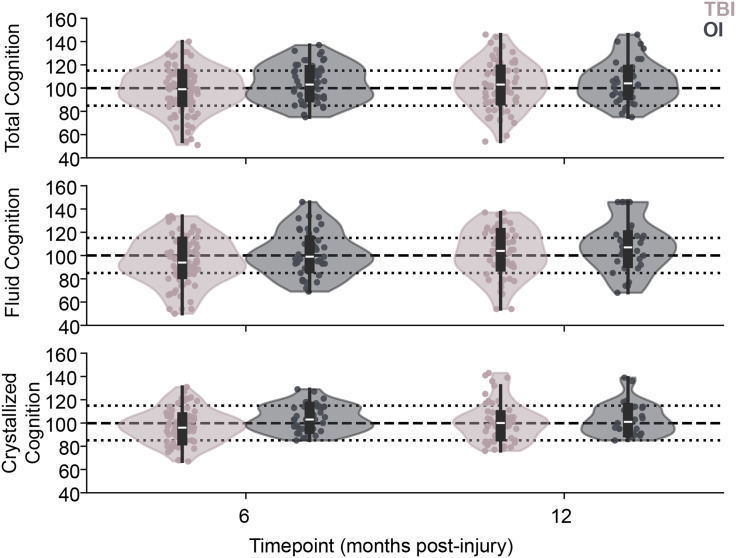
Models of Composite Cognition Scores over time for children with TBI (pink) versus OI (gray). No significant effects of injury group or group*time interaction were observed for Total Cognition (top), Fluid Cognition (middle), or for Crystallized Cognition (lower). Median and IQR are depicted by white line and the black bar in the violin plots, respectively.

Neither injury group nor the interaction of group and timepoint was associated with Total Cognition (group: F_1,174_ = 0.50, *p* = 0.50, *d* = 0.25; timepoint*group: F_1,174_ = 1.78, *p* = 0.185), Fluid Cognition (group: F_1,133_ = 1.10, *p* = 0.297, *d* = 0.30; timepoint*group: F_1,133_ = 0.04, *p* = 0.842), or Crystallized Cognition (F_1,135_ = 4.34, *p* = 0.039, *d* = 0.35; timepoint*group: F_1,135_ = 5.95, *p* = 0.017, Figure 2). Timepoint was significantly associated with Fluid Cognition, reflecting an increase in Fluid Cognition from six months to twelve months post-injury (F_1,137_ = 8.28, *p* = 0.005, *d* = 0.19, small effect). All group means fell in the ‘Average’ range. SES was significantly positively associated with all composite scores, indicating that higher SES (relative to our sample mean) was associated with better cognition (*p* < 0.001 for all models).

We also used linear mixed models to evaluate associations of injury group, timepoint, and the interaction of injury group and timepoint on individual test scores. On the individual tests, there were no significant associations of injury group or interaction of injury group*timepoint (*p* > 0.007 after Bonferroni correction) for any test (Supplemental Data). There were significant associations of timepoint for the Pattern Comparison (F_1,137_ = 10.93, *p* = 0.001) and Oral Reading Recognition (F_1,135_ = 7.971, *p* = 0.005), with greater age-corrected scores at twelve months than six months.

### Cognition by TBI severity

**Figure 3.**
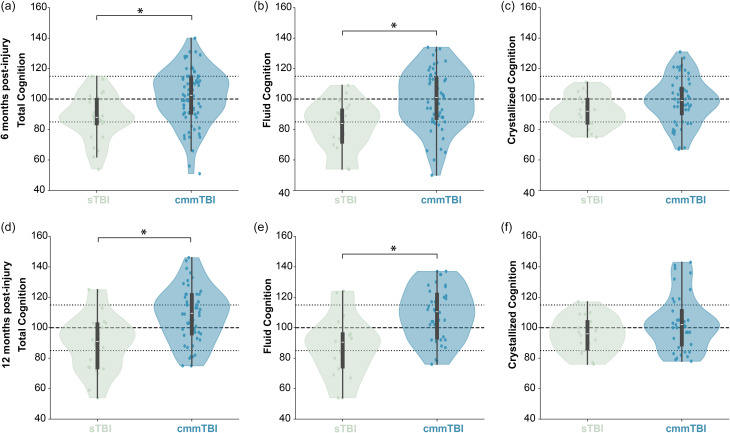
Cognition composite scores stratified by TBI severity. There were significant differences between children with sTBI (green) and cmmTBI (blue) for Total Cognition (A,D) and Fluid Cognition (B,E) composite scores at both six and twelve months post-injury. There were no significant differences in Crystallized Cognition (C,F) between severity groups. * indicates significant differences with Bonferroni correction for multiple comparisons (p < 0.008).

When stratified by severity, we found differences between TBI groups in Total Cognition and Fluid Cognition, but not Crystallized Cognition, at both timepoints (Figure 3). Children with sTBI performed significantly worse on Total Cognition than children with cmmTBI at six months (*p* = 0.012 partial *η*^2^ = 0.03, small–moderate effect) and twelve months post-injury (*p* = 0.002, partial *η*^2^ = 0.06, moderate effect). Mean scores for Total Cognition among children with sTBI were in the Average range at six and twelve months, whereas mean scores for children with cmmTBI were in the Average range at six months and the High Average range at twelve months post-injury. Similarly, children with sTBI performed significantly worse on Fluid Cognition than children with cmmTBI at six months (*p* = 0.004, partial *η*^2^ = 0.06, moderate effect) and twelve months post-injury (*p* < 0.001, partial *η*^2^ = 0.11, moderate–large effect). Mean scores for Fluid Cognition among children with sTBI were in the Low Average range at six and twelve months, whereas mean scores for children with cmmTBI were in the Average range at six and twelve months post-injury.

**Figure 4.**
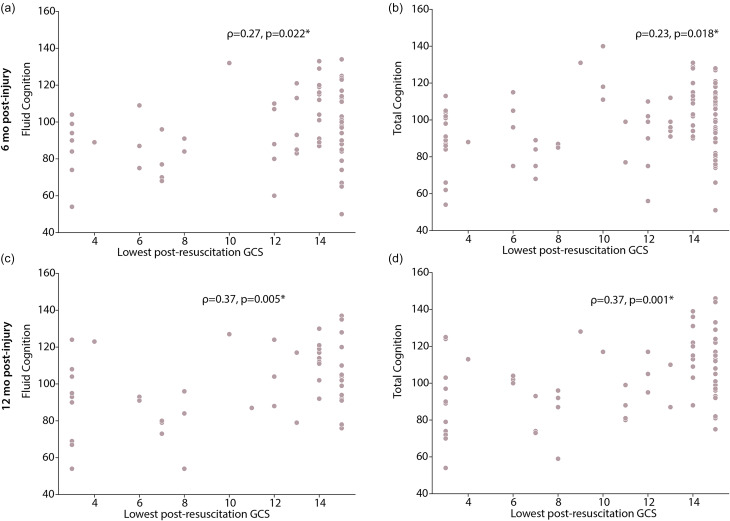
Spearman correlation with lowest post-resuscitation (LPR) GCS score for children with TBI. There were significant positive correlations of LPR GCS score with Fluid Cognition (A,C) and Total Cognition (B,D) at six months and twelve months post-injury. * indicates significant associations (p > 0.05).

Lowest post-resuscitation GCS score was significantly moderately positively correlated with Fluid Cognition at six months post-injury (*ρ* = 0.27, *p* = 0.022) and at twelve months post-injury (*ρ* = 0.37, *p* = 0.005, Figure 4). Additionally, we found moderate positive correlations of GCS score with Total Cognition at six months post-injury (*ρ* = 0.23, *p* = 0.018) and at twelve months post-injury (*ρ* = 0.37, *p* = 0.001). Correlations with Crystallized Cognition were not significant (*p* > 0.05).

## Discussion

On the NIH TB-CB, children with sTBI demonstrate lower cognitive performance (Total Cognition) than children with cmmTBI, as hypothesized. Furthermore, these differences are found in Fluid Cognition, but not Crystallized Cognition, again as hypothesized. However, there were not significant differences in cognitive performance between children with TBI and OI, contrary to our initial hypothesis.

Our finding of lower Total Cognition in children with sTBI as compared to children with cmmTBI is consistent with previous research. Previous studies show that children with mild TBI and complicated mild TBI generally demonstrate few deficits in cognitive performance after three months post-injury, while children with sTBI demonstrate consistently poorer cognitive performance compared to children with OI (Babikian & Asarnow, 2009; Chadwick et al., 2021; Keenan et al., 2018). Similarly, the previous study using the NIH TB-CB in children with mild TBI reported group differences between children with mild TBI versus OI with Fluid Cognition only ten days after injury, with no significant differences at three or six months after injury (Chadwick et al., 2021).

The lower scores in Fluid Cognition for children with sTBI compared to children with cmmTBI are also consistent with the previous studies indicating that Fluid Cognition is affected by TBI more than Crystallized Cognition (Chadwick et al., 2021; Tulsky et al., 2017). The low-average mean performance on Fluid Cognition in the sTBI group is typical of previous findings reporting neuropsychological outcomes in pediatric TBI (Anderson et al., 2012; Beauchamp et al., 2011; Catroppa & Anderson, 2003; Treble-Barna et al., 2017b). Additionally, the DSM-5^TM^ delineates neuropsychological performance 1 – 2 standard deviations below the mean as a modest cognitive impairment, suggesting the clinical importance of scores one standard deviation below the mean (*Diagnostic and Statistical Manual of Mental Disorders*, 2013). Among children with sTBI, 55% performed more than one standard deviation below the mean at six months and 45% performed more than one standard deviation below the mean at twelve months on Fluid Cognition. Among children with cmmTBI, only 14.8% at six months and 12.5% at twelve months performed more than one standard deviation below the mean on Fluid Cognition. The higher percentage of children with sTBI falling more than one standard deviation below the mean further substantiates the clinical significance of the differences in mean scores. The lack of differences in Crystallized Cognition among children with sTBI is also consistent with research indicating that Crystallized Cognition is generally spared after TBI (Chadwick et al., 2021; Tulsky, Carlozzi et al., 2017). We also demonstrated that TBI severity, as indexed by the GCS, was moderately positively correlated with both Total and Fluid Cognition up to twelve months post-injury. Taken together, these findings are consistent with prior studies showing that children with cmmTBI demonstrate recovery of Fluid Cognition relatively quickly compared to the sustained lower long-term Total and Fluid Cognition seen after sTBI.

In addition, the increase in Fluid Cognition composite scores for both the TBI and OI groups from six to twelve months post-injury suggest potential learning effects on the subtests constituting the Fluid Cognition composites. These findings may be driven by practice effects on the Pattern Comparison test (Supplemental Data).

The lack of differences between children with TBI and OI on Total Cognition and Fluid Cognition are contrary to our hypothesis. The lack of overall differences between children with TBI and OI are likely due to our sample distribution, reflecting the larger proportion of children with complicated mild TBI compared to sTBI in our sample. Based on the previous NIH-TB-CB findings in children with mild TBI compared to those with OI (Chadwick et al., 2021), we would not expect to detect significant differences in cognition past three months post-injury. Thus, we suggest a nuanced critique of the NIH TB-CB and recommendation for clinical utility. While the complete cognitive battery may not detect long-term impairments in children with complicated mild–moderate TBI, subtler differences in these children can be detected by using the NIH-TB-CB within the first three months of injury and evaluating reaction times on specific subtests (i.e., Flanker, Dimensional Change Card Sort).

Given the differences between children with sTBI and children with cmmTBI reported here, the NIH TB-CB may have clinical utility in under-resourced settings that lack the highly trained personnel, time, or materials needed for traditional comprehensive neuropsychological evaluation after TBI. The NIH TB-CB was designed to be a brief cognitive assessment that does not need to be administered by neuropsychologists or neuropsychological technicians and may provide a unique opportunity to broadly track cognitive recovery in children with TBI in these settings.

The study has several limitations. The high attrition from enrollment to six and twelve months follow-up appointments could potentially reduce the generalizability of our results to the broader pediatric TBI population. Attrition in our study was substantial for several reasons: (1) effects of the COVID-19 pandemic; (2) the relatively large catchment area of the UPMC Children’s Hospital of Pittsburgh and families’ preferences not to travel long distances for follow-up visits; (3) we began using version 3 of the NIH TB on June 6, 2024, so children who completed follow-ups after that date were not included in the current sample; and (4) families were hesitant to provide blood biospecimens, which were collected as part of the study. Despite the high attrition rate, the lack of significant differences in demographics and injury severity between children who did and did not return for follow-ups suggest a lack of selection bias in this sample. Because of the small number of participants with moderate TBI, data for children with complicated mild TBI and moderate TBI were combined for analyses. Aggregating data for complicated mild TBI and moderate TBI is consistent with approaches used in previous research characterizing neurobehavioral recovery after TBI in both children (Keenan et al., 2018, 2019; Levin et al., 2008) and adults (Tulsky et al., 2017). Additionally, although we attempted to complete the NIH TB-CB while children were hospitalized acutely after injury, acute assessments were very limited in our sample, largely due to time constraints of the hospital stay. Furthermore, locating an environment suitable for testing on the typical acute trauma unit is difficult. However, previous studies found that using the NIH TB-CB is feasible in an inpatient rehabilitation hospital setting and day treatment rehabilitation facilities, where frequent interruptions for nursing care, therapies, etc., are reduced (Watson et al., 2020). Furthermore, prior studies were able to evaluate the NIH TB-CB in a large sample of children with mild TBI as early as 10 days post-injury (Chadwick et al., 2021). Including a larger sample of acute data would provide more insight into the trajectories of recovery in children with TBI, as measured by the NIH TB-CB.

We also acknowledge the limitations of classifying TBI severity by GCS score alone, as noted by the recent National Institute of Neurological Disorders and Stroke TBI Classification and Nomenclature Workshop. The workshop yielded recommendations to expand TBI classification to include additional clinical assessments, neuroimaging, blood-based biomarkers and psychological and environmental modifiers (Bazarian et al., 2025; Bragge et al., 2025; Corrigan et al., 2025; Mac Donald et al., 2025; Manley et al., 2025; Menon et al., 2025; Nelson et al., 2025). While these recommendations were predominantly geared towards adults, studies evaluating reference ranges of biomarkers and neuroimaging in pediatric TBI are ongoing (Marzano et al., 2022; Mondello et al., 2016; Munoz Pareja et al., 2022; Reisner et al., 2024).

Furthermore, our cohort contains children across a wide age range, spanning ages 3 to 18. Recent studies have reported that 3-year-olds may struggle to perform three of the four tests on the Early Cognition version of the NIH TB-CB (Becker et al., 2023). In our sample, two 3-year-old children with sTBI could not complete the Early Childhood NIH TB-CB and were excluded from analysis. Of the included 3-year-olds, we had an even distribution across injury and severity groups (*n* = 1 sTBI, *n* = 3 cmmTBI, *n* = 3 OI). All the 3-year-old children who completed the test scored within the Low Average to Extremely High performance ranges and were not outliers in our dataset (> 1.5*IQR of Total Cognition composite scores). In future studies, however, best practices suggest that only children aged 4+ should be prospectively included in the study design when using the NIH TB-CB.

While the association of SES with cognition has been well studied, recent evidence suggests that the interaction between SES and biological variables (e.g. side of hemispheric injury, genomics, neural imaging) may contribute to this relationship (Cohen-Zimerman et al., 2019; Noble et al., 2012; Raizada et al., 2008; Schneider et al., 2024; Shi et al., 2024). Future studies evaluating biological factors (biomarkers, epigenetics, imaging) and their role in the relationship between SES and cognition in pediatric TBI could shed additional light on mechanisms of heterogeneity underlying these associations. The findings here also support the critical importance of continuing to include SES as a covariate in models of cognition after TBI.

## Conclusion

Here we provide evidence that the NIH TB-CB detects lower cognitive performance in children with sTBI at six and twelve months post-injury, but not children with cmmTBI, consistent with previous research on cognitive outcomes of pediatric TBI. The findings largely reflect differences in Fluid Cognition, which are more pronounced in children with sTBI. In settings with inadequate time, resources, or personnel to complete comprehensive neuropsychological testing, our findings suggest the NIH TB-CB may be a suitable option for longitudinal monitoring of cognition in children with TBI in research and clinical contexts.

## Supporting information

Petersen et al. supplementary material Petersen et al. supplementary material

## Supplementary material

The supplementary material for this article can be found at https://doi.org/10.1017/S135561772510146X.
