# Supplementary material for: Evaluating cognitive performance using the National Institutes of Health Toolbox Cognitive Battery in children with traumatic brain injury
Source: J Int Neuropsychol Soc. 2025 Oct 24;31(5-6):396–405. doi: 10.1017/S135561772510146X (PMC12990185; doi:10.1017/S135561772510146X)
Supplement: Petersen et al. supplementary material [file S135561772510146Xsup001.docx]

**Supplementary Data**

**Table 1.** Participant demographics for participants excluded from analysis (enrolled only, no follow-up outcomes) versus participants included in analysis (enrolled with 6 or 12 month follow-up outcomes). We assessed differences between participants enrolled only and participants with 6 or 12 month follow-up outcomes separately for OI and TBI groups. We used a t-test for normally distributed continuous data (SES composite z-score), a Mann-Whitney U Test for non-normally distributed continuous data and a chi-squared test for categorical data. Race was categorized as white vs. all other race groups (combined due to small cell sizes). * indicates significant differences (p<0.05).

|  | **OI** | | | **TBI** | | |
| --- | --- | --- | --- | --- | --- | --- |
|  | Enrolled only (n=86) | Enrolled with 6 or 12 month follow-up  (n=60) | Differences between enrolled and follow-up groups,  p-value | Enrolled only  (n= 111) | Enrolled with 6 or 12 month follow-up  (n=120) | Differences between enrolled and follow-up groups,  p-value |
| Age at Injury in yrs (median [IQR]) | 10.82 [7.45, 15.63] | 10.61 [6.86, 14.24] | 0.27 | 11.23 [5.53, 14.52] | 10.88 [6.03, 14.42] | 0.84 |
| Lowest post-resuscitation GCS (Median [IQR]) | --- | --- | --- | 14.0 [8.0, 15.0] | 14.0 [8.0, 15.0] | 0.59 |
| SES Composite Z-score (Mean (SD)) | -0.07 (0.72) | 0.16 (0.83) | 0.08 | -0.02 (0.75) | -0.02 (0.80) | 0.99 |
| Child Race, n (%) |  |  | 1.0 |  |  | 1.0 |
| White | 72 (83.7) | 48 (77.4) |  | 71 (64.0) | 106 (88.3) |  |
| Black or African American | 7 (8.1) | 8 (12.9) |  | 14 (12.6) | 11 (9.2) |  |
| Asian | 0 (0) | 2(3.2) |  | 1 (0.09) | 1 (0.08) |  |
| Other | 3 (3.5) | 0 (0) |  | 1 (0.09) | 0 (0) |  |

**Table 2.** Participant demographics by follow-up visit. We assessed differences between injury groups (TBI vs OI) using an ANOVA for normally distributed continuous data (SES composite z-score), a Kruskal Wallis Test for non-normally distributed continuous data and a chi-squared test for categorical data. Race was categorized as white vs. all other race groups (combined due to small cell sizes). * indicates significant differences (p<0.05).

|  | **OI** | | | | **TBI** | | | |
| --- | --- | --- | --- | --- | --- | --- | --- | --- |
|  | 6 month follow-up  (n=17) | 12 month follow-up  (n=9) | Both 6 and 12 month follow-up  (n=34) | Differences between follow-up groups,  p-value | 6 month follow-up only  (n=36) | 12 month follow-up only  (n=12) | Both 6 and 12 month follow-up  (n=72) | Differences between follow-up groups,  p-value |
| Age at Injury in yrs (median [IQR]) | 10.58 [6.01, 15.05] | 8.98 [5.24, 9.72] | 11.92 [7.18, 14.63] | 0.11 | 10.81 [6.72, 13.91] | 10.09 [5.24, 14.41] | 11.30 [6.04, 14.60] | 0.88 |
| Lowest post-resuscitation GCS (Median [IQR]) | --- | --- | --- | --- | 14.0 [12.0, 15.0] | 15.0 [11.75, 15.0] | 13.0 [6.75, 15.0] | 0.050 |
| SES Composite Z-score (Mean (SD)) | 0.01 (0.83) | -0.37 (0.47) | 0.03 (0.81) | 0.60 | 0.10 (0.80) | -0.05 (0.84) | 0.25 (0.85) | 0.26 |
| Child Race, n (%) |  |  |  | 1.0 |  |  |  | 1.0 |
| White | 14 (82.4) | 7 (77.8) | 27 (79.4) |  | 32 (88.9) | 10 (83.3) | 62 (86.1) |  |
| Black or African American | 2 (11.8) | 1 (11.1) | 5 (14.7) |  | 3 (8.3) | 1 (8.3) | 7 (9.7) |  |
| Asian | 0 (0) | 1 (11.1) | 1 (2.9) |  | 0 (0) | 0 (0) | 1 (1.4) |  |
| Other | 0 (0) | 0 (0) | 0 (0) |  | 0 (0) | 0 (0) | 0 (0) |  |

**Table 3.** Linear mixed model results for Picture Vocabulary Age-Corrected scores. The Picture Vocabulary test measures vocabulary comprehension (Weintraub et al., 2013). Age-corrected scores are adjusted to age-matched normative scores, with a mean (standard deviation) of 100 (15). No significant association was found between group (TBI vs OI), timepoint, or the interaction between group and timepoint. SES was significantly positively associated with Picture Vocabulary scores. (Statistical significance was determined by α=0.007 after Bonferroni correction.)

| Variable | Picture Vocabulary  (n=179) | | | |
| --- | --- | --- | --- | --- |
|  | Estimate | 95% CI | F | p-value |
| Intercept | 99.13 | [95.91, 102.34] | 8587.80 | <0.001* |
| Group (ref=TBI) | 1.88 | [-3.00, 6.76] | 3.38 | 0.068 |
| Timepoint (ref=12M) | 0.16 | [-2.49, 2.80] | 3.40 | 0.067 |
| Sex (ref=M) | -0.17 | [-4.20, 3.86] | 0.007 | 0.933 |
| Group*Timepoint (ref=12M & TBI) | 3.96 | [-0.62, 8.53] | 2.92 | 0.090 |
| SES | 5.27 | [2.90, 7.64] | 19.23 | <0.001* |

**Table 4.** Linear mixed model results for Flanker Age-Corrected scores. The Flanker test measures inhibitory control and attention, with better scores indicating better performance relative to age-matched norms (Weintraub et al., 2013). SES was significantly positively associated with Flanker scores, while group, sex, timepoint, and group-by-timepoint interaction were not significant. (Statistical significance was determined by α=0.007 after Bonferroni correction.)

| Variable | Flanker  (n=179) | | | |
| --- | --- | --- | --- | --- |
|  | Estimate | 95% CI | F | p-value |
| Intercept | 93.98 | [90.60, 97.36] | 6960.11 | <0.001* |
| Group (ref=TBI) | 0.04 | [-5.13, 5.20] | 0.09 | 0.772 |
| Timepoint (ref=12M) | -0.37 | [-3.35, 2.61] | 0.63 | 0.430 |
| Sex (ref=M) | 0.98 | [-3.18, 5.14] | 0.22 | 0.643 |
| Group*Timepoint  (ref=12M & TBI) | -1.34 | [-6.52, 3.85] | 0.26 | 0.611 |
| SES | 5.50 | [3.06, 7.94] | 19.77 | <0.001* |

**Table 5.** Linear mixed model results for List Sorting Working Memory Age-Corrected scores. The List Sorting Working Memory test measures working memory using a sequencing task with visual and auditory stimuli (Tulsky et al, 2014). SES was significantly positively associated with List Sorting Working Memory scores, while group, sex, timepoint, and group-by-timepoint interaction were not significant. (Statistical significance was determined by α=0.007 after Bonferroni correction.)

| Variable | List Sorting Working Memory  (n=134) | | | |
| --- | --- | --- | --- | --- |
|  | Estimate | 95% CI | F | p-value |
| Intercept | 100.20 | [96.39, 104.01] | 6256.92 | <0.001* |
| Group (ref=TBI) | 3.83 | [-1.94, 9.61] | 2.69 | 0.103 |
| Timepoint (ref=12M) | 0.24 | [-3.41, 3.88] | 0.04 | 0.847 |
| Sex (ref=M) | -5.03 | [-9.79, -0.28] | 4.37 | 0.038 |
| Group*Timepoint  (ref=12M & TBI) | 0.12 | [-5.98, 6.23] | 0.00 | 0.968 |
| SES | 6.72 | [4.04, 9.41] | 24.49 | <0.001* |

**Table 6.** Linear mixed model results for Dimensional Change Card Sort Age-Corrected scores. The Dimensional Change Card Sort test assesses executive function by testing children on their ability to sort cards by shape or color (Doebel & Zelazo 2015). SES was significantly positively associated with Dimensional Change Card Sort scores, while group, sex, timepoint, and group-by-timepoint interaction were not significant. (Statistical significance was determined by α=0.007 after Bonferroni correction.)

| Variable | Dimensional Change Card Sort  (n=178) | | | |
| --- | --- | --- | --- | --- |
|  | Estimate | 95% CI | F | p-value |
| Intercept | 100.79 | [97.19, 104.38] | 6841.52 | <0.001* |
| Group (ref=TBI) | 0.32 | [-5.13, 5.77] | 0.20 | 0.653 |
| Timepoint (ref=12M) | -3.29 | [-6.45, -0.14] | 3.54 | 0.62 |
| Sex (ref=M) | -1.82 | [-6.23, 2.60] | 0.66 | 0.418 |
| Group*Timepoint  (ref=12M & TBI) | 1.42 | [-4.01, 6.86] | 0.27 | 0.605 |
| SES | 6.72 | [4.13, 9.32] | 26.20 | <0.001* |

**Table 7.** Linear mixed model results for Pattern Comparison Processing Speed (PCPS) Age-Corrected scores. Higher Pattern Comparison Processing Speed scores indicate a greater number of correct answers in 90 seconds and therefore a better processing speed (Carlozzi, Tulsky, et al., 2014). SES was significantly positively associated with Pattern Comparison Processing Speed scores, while timepoint was significantly negatively associated (indicating children at 6 months had slower processing speed than at 12 months). Group, sex, and the group-by-timepoint interaction were not significant. (Statistical significance was determined by α=0.007 after Bonferroni correction.)

| Variable | Pattern Comparison Processing Speed  (n=137) | | | |
| --- | --- | --- | --- | --- |
|  | Estimate | 95% CI | F | p-value |
| Intercept | 106.87 | [100.48, 113.25] | 2146.08 | <0.001* |
| Group (ref=TBI) | 1.13 | [-8.25, 10.51] | 0.01 | 0.926 |
| Timepoint (ref=12M) | -5.81 | [-10.56, -1.07] | 10.93 | 0.001* |
| Sex (ref=M) | -3.36 | [-11.72, 5.01] | 0.63 | 0.429 |
| Group*Timepoint  (ref=12M & TBI) | -1.48 | [-9.35, 6.40] | 0.14 | 0.710 |
| SES | 8.42 | [3.63, 13.21] | 12.09 | <0.001* |

**Table 8.** Linear mixed model results for Picture Sequence Memory Age-Corrected scores. The Picture Sequence Memory Test examines episodic memory by presenting a fixed sequence of objects (Dikmen, Bauer, et al., 2014). SES was significantly positively associated with Picture Sequence Memory scores, while group, sex, timepoint, and group-by-timepoint interaction were not significant. (Statistical significance was determined by α=0.007 after Bonferroni correction.)

| Variable | Picture Sequence Memory  (n=179) | | | |
| --- | --- | --- | --- | --- |
|  | Estimate | 95% CI | F | p-value |
| Intercept | 102.62 | [98.493, 106.75] | 5855.18 | <0.001* |
| Group (ref=TBI) | -0.12 | [-6.43, 6.20] | 0.001 | 0.974 |
| Timepoint (ref=12M) | -0.62 | [-4.48, 3.24] | 0.06 | 0.803 |
| Sex (ref=M) | 1.38 | [-3.59, 6.36] | 0.30 | 0.584 |
| Group*Timepoint  (ref=12M & TBI) | 0.40 | [-6.25, 7.05] | 0.01 | 0.906 |
| SES | 7.40 | [4.48, 10.32] | 25.01 | <0.001* |

**Table 9.** Linear mixed model results for Oral Reading Recognition Age-Corrected scores. The Oral Reading Recognition Test evaluates reading decoding proficiency and crystallized intelligence (Weintraub et al., 2013). SES was significantly positively associated with Oral Reading Recognition scores, while timepoint was significantly negatively associated, indicating children had better reading decoding proficiency at 12M. Group, sex, and group-by-timepoint interaction were not significant. (Statistical significance was determined by α=0.007 after Bonferroni correction.)

| Variable | Oral Reading Recognition  (n=135) | | | |
| --- | --- | --- | --- | --- |
|  | Estimate | 95% CI | F | p-value |
| Intercept | 99.13 | [95.50, 102.76] | 5995.22 | <0.001* |
| Group (ref=TBI) | 1.96 | [-3.37, 7.29] | 2.28 | 0.134 |
| Timepoint (ref=12M) | -4.76 | [-7.27, -2.24] | 8.21 | 0.005* |
| Sex (ref=M) | 0.86 | [-3.98, 5.71] | 0.12 | 0.726 |
| Group*Timepoint  (ref=TBI, 12M) | 3.37 | [-0.90, 7.63] | 2.46 | 0.121 |
| SES | 7.32 | [4.56, 10.08] | 27.61 | <0.001* |

**Table 10.** Linear mixed model results for Cognition Fluid Composite Age-Corrected scores. SES was significantly positively associated with Cognition Fluid Composite scores, while timepoint was significantly negatively associated. Group, sex, and the group-by-timepoint interaction were not significant. (Statistical significance was determined by α= 0.017 after Bonferroni correction.)

| Variable | Cognition Fluid Composite  (n=133) | | | |
| --- | --- | --- | --- | --- |
|  | Estimate | 95% CI | F | p-value |
| Intercept | 99.28 | [94.42, 104.15] | 3249.86 | <0.001* |
| Group (ref=TBI) | 3.68 | [-3.45, 10.80] | 1.10 | 0.297 |
| Timepoint (ref=12M) | -3.73 | [-7.01, -0.45] | 8.28 | 0.005* |
| Sex (ref=M) | -2.60 | [-9.10, 3.90] | 0.63 | 0.430 |
| Group*Timepoint  (ref=TBI, 12M) | -0.56 | [-6.09, 4.98] | 0.04 | 0.842 |
| SES | 10.67 | [6.99, 14.36] | 32.79 | <0.001* |

**Table 11.** Linear mixed model results for Cognition Crystallized Composite Age-Corrected scores. SES was significantly positively associated with Cognition Crystallized Composite scores, while group, sex, timepoint, and group-by-timepoint interaction were not significant. (Statistical significance was determined by α= 0.017 after Bonferroni correction.)

| Variable | Cognition Crystallized Composite  (n=135) | | | |
| --- | --- | --- | --- | --- |
|  | Estimate | 95% CI | F | p-value |
| Intercept | 99.14 | [95.78, 102.50] | 6721.45 | <0.001* |
| Group (ref=TBI) | 2.76 | [-2.14, 7.67] | 4.34 | 0.039 |
| Timepoint (ref=12M) | -2.98 | [-4.92, -1.03] | 1.29 | 0.259 |
| Sex (ref=M) | -0.41 | [-5.03, 4.21] | 0.03 | 0.862 |
| Group*Timepoint  (ref=12M & TBI) | 4.06 | [0.75, 7.37] | 5.95 | 0.017 |
| SES | 7.15 | [4.52, 9.78] | 28.93 | <0.001* |

**Table 12.** Linear mixed model results for Total Cognition Age-Corrected scores. SES was significantly positively associated with Total Cognition scores, while group, sex, timepoint, and group-by-timepoint interaction were not significant. (Statistical significance was determined by α= 0.017 after Bonferroni correction.)

| Variable | Total Cognition  (n=174) | | | |
| --- | --- | --- | --- | --- |
|  | Estimate | 95% CI | F | p-value |
| Intercept | 100.66 | [97.02, 104.30] | 6191.57 | <0.001* |
| Group (ref=TBI) | 0.17 | [-5.27, 5.60] | 0.50 | 0.481 |
| Timepoint (ref=12M) | -3.25 | [-5.85, -0.65] | 2.11 | 0.149 |
| Sex (ref=M) | -0.26 | [-4.96, 4.44] | 0.01 | 0.912 |
| Group*Timepoint  (ref=12M & TBI) | 3.11 | [-1.51, 7.74] | 1.78 | 0.185 |
| SES | 11.0 | [8.26, 13.75] | 62.59 | <0.001* |

**Bibliography**

Carlozzi NE, Tulsky DS, Chiaravalloti ND, et al. NIH Toolbox Cognitive Battery (NIHTB-CB): the NIHTB Pattern Comparison Processing Speed Test. J Int Neuropsychol Soc. 2014;20(6):630-641. doi:10.1017/S1355617714000319

Dikmen SS, Bauer PJ, Weintraub S, et al. Measuring episodic memory across the lifespan: NIH Toolbox Picture Sequence Memory Test. J Int Neuropsychol Soc. 2014;20(6):611-619. doi:10.1017/S1355617714000460

Doebel S, Zelazo PD. A meta-analysis of the Dimensional Change Card Sort: Implications for developmental theories and the measurement of executive function in children. Dev Rev. 2015;38:241-268. doi:10.1016/j.dr.2015.09.001

Tulsky DS, Carlozzi N, Chiaravalloti ND, et al. NIH Toolbox Cognition Battery (NIHTB-CB): list sorting test to measure working memory. J Int Neuropsychol Soc. 2014;20(6):599-610. doi:10.1017/S135561771400040X

Weintraub, S., Bauer, P. J., Zelazo, P. D., Wallner-Allen, K., Dikmen, S. S., Heaton, R. K., … Gershon, R. C. (2013). I. Nih Toolbox Cognition Battery (cb): Introduction and Pediatric Data. Monographs of the Society for Research in Child Development, 78(4), 1–15. doi:10.1111/mono.12031
